# Supplementary material for: Hierarchically self-assembled hexagonal honeycomb and kagome superlattices of binary 1D colloids
Source: Nat Commun. 2017 Aug 25;8:360. doi: 10.1038/s41467-017-00512-9 (PMC5572454; doi:10.1038/s41467-017-00512-9)
Supplement: Supplementary file 1 — Supplementary Information [file 41467_2017_512_MOESM1_ESM.pdf]

SI GUIDE

File Name: Supplementary Information
Description: Supplementary Figures, Supplementary Tables, Supplementary Notes, and Supplementary References.

File Name: Peer Review File

Supplementary Note 1: SANS Form Factor Analysis of *p*-C_nTVB.

The SANS intensities of *p*-C_nTVB in D_2O (0.1% by weight with 0.01 M NaCl to screen the electrostatic interaction between *p*-C_nTVBs) were analyzed using a rigid cylindrical particle form factor. For a very dilute solution of particles in which the interparticle interaction is negligible, the scattering intensity can be simplified as

$$I(q) = n_\mathrm{p} P(q) + b \tag{1}$$

where n_p is the number density of particles, $P(q)$ is the particle form factor, and b is the incoherent scattering. The rigid cylindrical particle form factor is given as[1–3]

$$P(q) = \int_0^{\frac{\pi}{2}} f^2(q,\alpha) \sin\alpha \, \mathrm{d}\alpha \tag{2}$$

$$f(q,\alpha) = 2(\rho_\mathrm{cyl} - \rho_\mathrm{solv}) V j_0\left(\frac{qL\cos\alpha}{2}\right)\frac{J_1(qR\sin\alpha)}{(qR\sin\alpha)} \tag{3}$$

$$j_0 = \sin x / x \tag{4}$$

where V is the particle volume and, ρ_cyl and ρ_solv are neutron scattering length densities of the particles and solvent, respectively. $J_1(x)$ is the first-order Bessel function and α is the angle between the cylinder axes and the scattering vector (q). The scattering length density (SLD) of D_2O is 6.33×10^{-6} Å^{-2}. The calculated scattering length densities and fitting results of radius and length of *p*-C_nTVB for n = 10, 12, 14, and 16 are summarized in Supplementary Table 1.

Supplementary Table 1. **Calculated SLDs, fitted diameters, and lengths of *p*-C_nTVB**.

n	SLD (10^{-6} Å^{-2})	Diameter (nm)	Polydispersity of diameter	Length (nm)
10	1.061	2.52	0.03	39
12	0.903	2.96	0.02	23
14	0.651	3.44	0.06	29
16	0.478	3.96	0.14	69

Supplementary Note 2: Phase Transition Temperature Depending on *n*.

For *p*-C_nTVB/$C_{12}E_5$/water (10/45/55) samples, the transition temperature from the isotropic to hexagonal phases decreases (for n = 10 and 12) or increases (for n = 14 and 16) compared to that of $C_{12}E_5$/water (45/55). The decrease of transition temperature for n = 12 is not clearly visible in Fig. 3. However, when the concentration of *p*-C_{12}TVB in the mixture is increased to (15/45/55), the transition temperature was decreased by 2.5 °C compared to that of $C_{12}E_5$/water (45/55). The transition from the hexagonal to isotropic phases (i.e. melting of hexagonal lattice) is induced by the increased undulation of cylinders with temperature.[4] The *p*-C_nTVBs may have two competing contributions to the transition temperature. The rigid nature of *p*-C_nTVBs may restrict the undulation of $C_{12}E_5$ cylinders, increasing the transition temperature. On the other hand, the smaller diameter of *p*-C_nTVBs than that of $C_{12}E_5$ cylinders allows more free volume for $C_{12}E_5$ cylinders for undulation, decreasing the transition temperature. For *p*-C_nTVBs with n = 14 and 16 (diameter ratio of 0.79 and 0.91, respectively, and rigid), the first effect may be more pronounced than the second effect, resulting in the increase of transition temperature. For *p*-C_nTVBs with n = 10 and 12 (diameter ratio of 0.59 and 0.68, respectively, and less rigid), the second effect may be more pronounced than the first effect, resulting in the decrease of transition temperature.

Supplementary Table 2. **Peak positions and corresponding lattice parameters of *p*-C_nTVB/$C_{12}E_5$/water (*n* = 10, 12, 14, and 16) at the mixing ratio of 10/45/55.**

n	Peak position (q, nm^{-1})							Lattice Parameter (nm)
	1st	2nd	3rd	4th	5th	6th	7th	
	1	√3	2	√7	3	√12	√13	
10	0.75	1.30	1.49	1.98	2.24	2.59	2.69	9.71
12	0.75	1.30	1.49		2.24	2.58		9.71
14	0.74	1.29			2.22	2.56		9.77
16	0.76	1.31			2.27	2.62		9.59

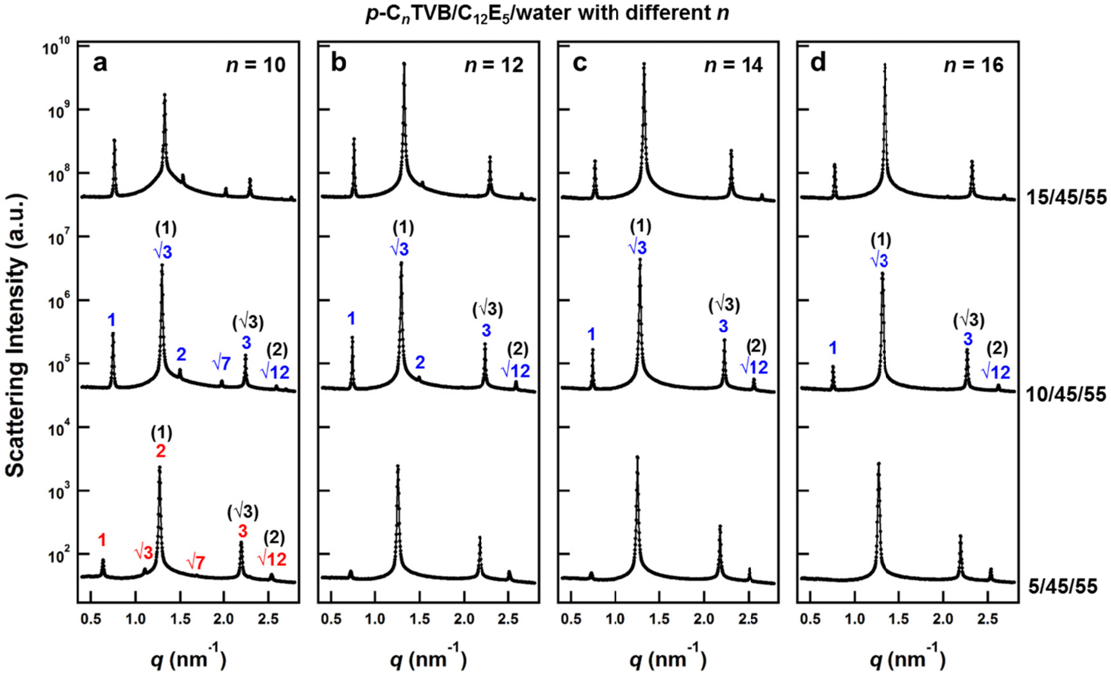

Supplementary Figure 1. **Effects of diameter and mixing ratios between *p*-C*$_n$*TVB and $C_{12}E_5$ cylinders** SAXS intensities of (a) *p*-C_{10}TVB/$C_{12}E_5$/water, (b) *p*-C_{12}TVB/$C_{12}E_5$/water, (c) *p*-C_{14}TVB/$C_{12}E_5$/water, and (d) *p*-C_{16}TVB/$C_{12}E_5$/water at different mixing ratios. All the measurements were performed at 6 °C. Scattering intensities are shifted vertically for visual clarity.

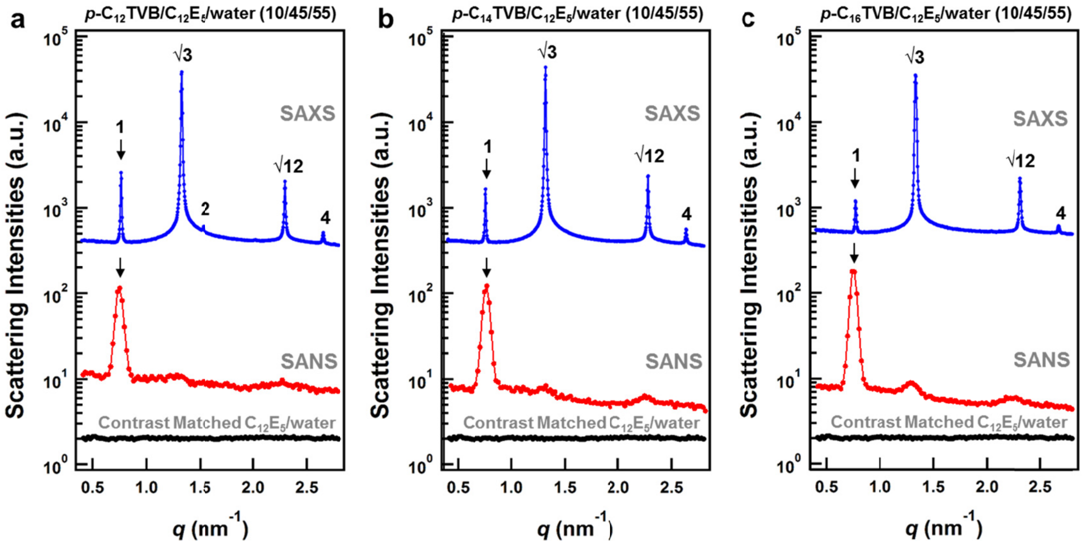

Supplementary Figure 2. **Contrast Matched SANS measurements.** SANS intensities of the partially deuterated *p*-C_nTVB (n = 12, 14, and 16) mixed with the contrast-matched $C_{12}E_5$/water at the mixing ratio of (10/45/55). SAXS intensities are provided for comparison. All the measurements were performed at 6 °C. Scattering intensities are shifted vertically for visual clarity.

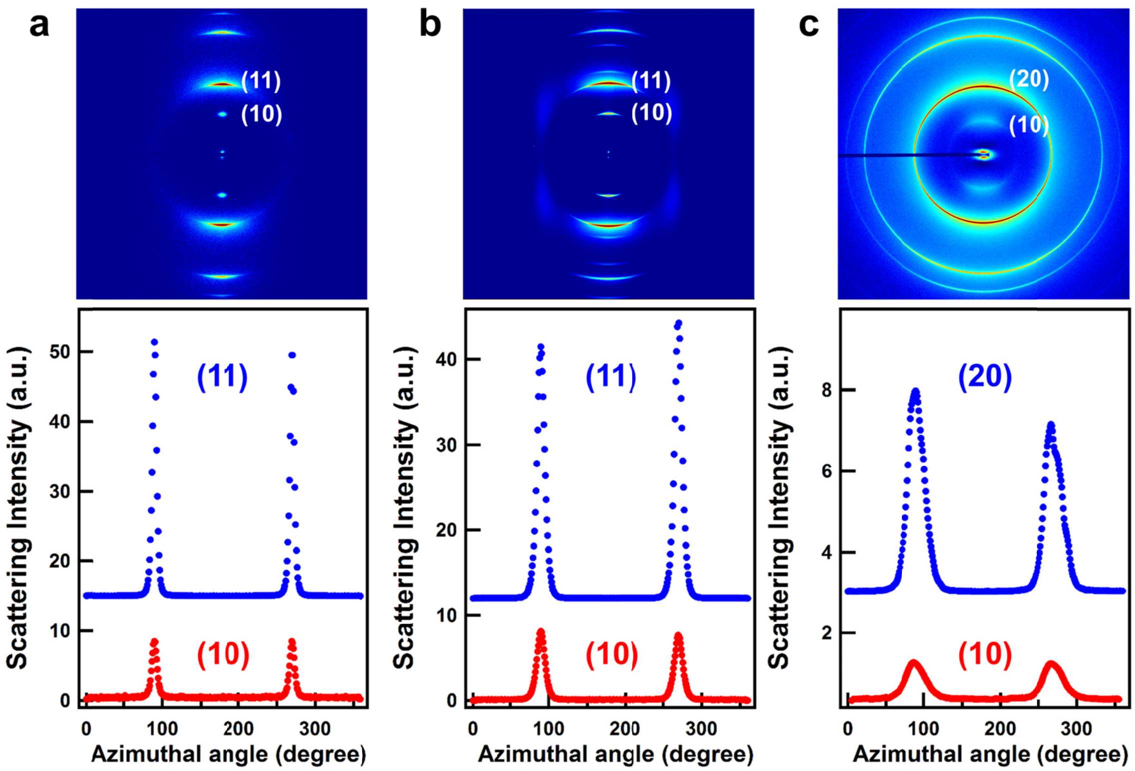

Supplementary Figure 3. SAXS measurements for shear induced alignment along the radial direction. The SAXS measurements along the radial direction for the samples of (a) *p*-C_{14}TVB/$C_{12}E_5$/water (10/45/55), (b) *p*-C_{10}TVB/$C_{12}E_5$/water (10/45/55), and (c) *p*-C_{10}TVB/$C_{12}E_5$/water (5/45/55) under an oscillatory shear with a shear stress of 500 Pa and a shear frequency of 5 Hz. The 2D patterns and their corresponding azimuthally averaged intensities are presented in upper and lower parts, respectively.

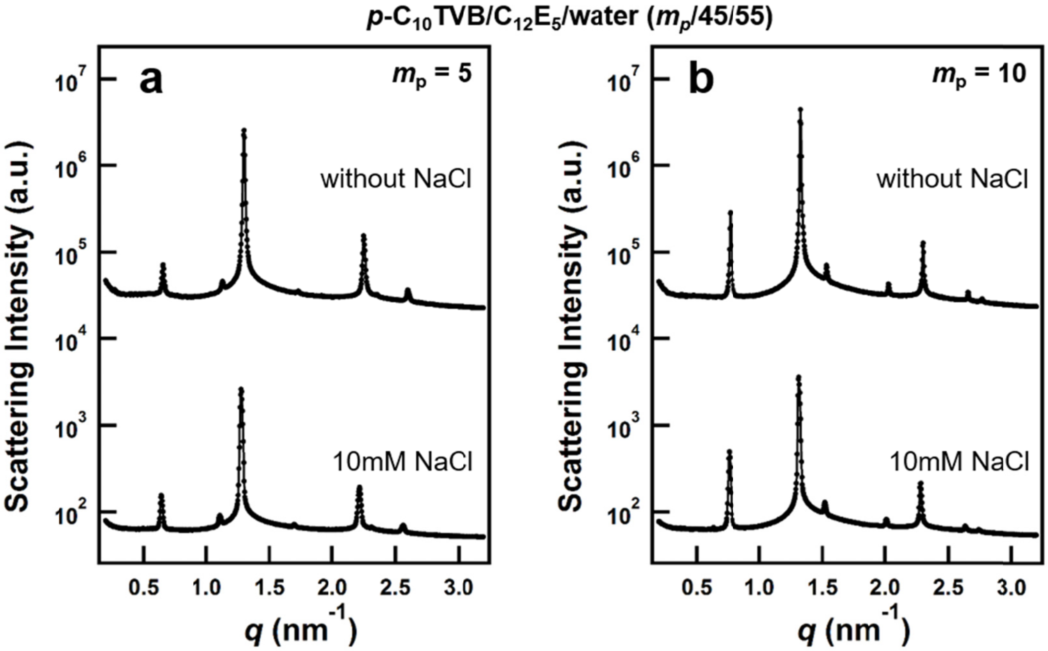

Supplementary Figure 4. Effects of salt on the formation of binary superlattices. SAXS intensities of *p*-C_{10}TVB/$C_{12}E_5$/water samples with and without 10 mM of NaCl for two different mixing ratios, (a) (5/45/55) and (b) (10/45/55).

Supplementary Note 3: Theoretical Model.

We employ a mean-field theory to calculate the free energy (F) of our systems and understand the thermodynamics of the phase transition. Since we are interested in the crystallization of p-C_nTVBs and $C_{12}E_5$ in a lateral direction, we model p-C_nTVBs and $C_{12}E_5$ as two-dimensional hard discs A and B of different effective diameters, respectively. In this study, the values of diameters are tuned so that the interaction between p-C_nTVBs and $C_{12}E_5$ is incorporated into the effective diameters. The diameter of disc B ($C_{12}E_5$) is used as a length unit in this study.

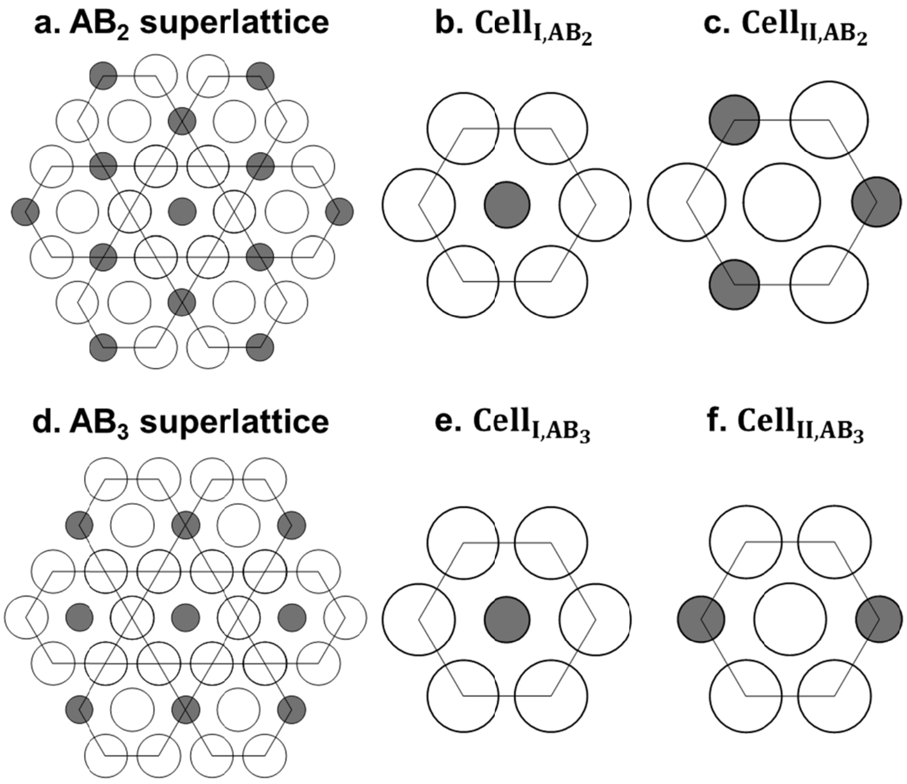

Supplementary Figure 5. Schematics of theoretical cell model. (a) AB_2 superlattice phase where grey and empty circles represent A and B discs, respectively, and (b) and (c) two different types of cells of AB_2 superlattice phase. (d) AB_3 superlattice phase, and (e) and (f) two different types of cells of AB_3 superlattice phase.

We consider three different phases that are observed in experiments: (1) AB_3 superlattice phase, (2) AB_2 superlattice phase, and (3) random lattice phase. p-C_nTVBs (disc A) and $C_{12}E_5$ (disc B) in all three phases are located at hexagonal lattice points (Supplementary Fig.

5a and 5d). In AB_3 and AB_2 superlattices, A and B form crystals in regular fashions with the mole fraction of A in the superlattice phase (x_S) of 0.25 and 0.33, respectively. On the other hand, in case of a random phase, A and B are randomly distributed on the hexagonal lattice points with the mole fraction of A in the random phase (x_R) ranged from 0 to 1.

Our experiments suggest that superlattice phases would coexist with a random phase. Note that the value of x_S for superlattice phases is fixed at either 0.25 or 0.33. However, the mole fraction of p-C_nTVBs in our experiments ranges from 0.11 to 0.43, and the superlattice phases are still observed. This indicates that while some A discs form superlattices, the rest of A discs might still exist and form a random phase with a particular value of x_R. The free energy (F) of our systems, then, needs to be represented as a linear combination of both the free energies *per site* (F_S and F_R) of the superlattice phase and the random phase.

$$F = (1-\phi)F_S + \phi F_R(x_R) \quad (5)$$

Here, ϕ is an area fraction (the number fraction of sites) of the random phase. While F_R is a function of x_R, F_S is a constant for given values of disc diameters and the lattice dimensions. Note that the effect of the interface between the superlattice phase and the random phase is ignored.

If the two phases were to coexist, the chemical potential of two phases should be identical. We, therefore, need to determine the value of x_R, for which the chemical potential of A discs in the random phase ($\mu_{A,R}$) is equal to the chemical potential of A discs in the superlattice phase ($\mu_{A,S}$), i.e., $\mu_{A,S} = \mu_{A,R}(x_R)$. Once the value of x_R is determined, the area fraction of the random phase (ϕ) can be estimated for a given mole fraction of A in the system (x), i.e., $\phi = \frac{x-x_S}{x_R-x_S}$. Here, x_S = 0.25 or 0.33 for AB_3 and AB_2 superlattice phases, respectively.

We employed the cell theory to calculate both the free energies per site (F_S and F_R) and the chemical potentials ($\mu_{A,S}$ and $\mu_{A,R}$).[7,8] The cell theory has been employed successfully to estimate the free energy per site of the crystalline solids.[9,10] In the cell theory, we consider all different types of cells for each phase. Each of AB_3 and AB_2 superlattice phases consists of 2 types of cells (Supplementary Fig. 5b, 5c, 5e, and 5f). In case of the random phase, on the other hand, there are 128 different cells. As a first step to calculate F_S and F_R, we need to calculate the partition function $Q = Q(l, \sigma)$ of each cell as a function of the hexagonal lattice dimension (l) and the diameter ratio (σ) of A to B discs. In this study, we use l = 1.05. We employ the Widom method to calculate $Q(l, \sigma)$.[11] We place a test disc (identical to a disc A) at a random position in each cell and check if the test disc overlaps with any other disc at the

lattice points of the cell. We repeat the above procedure 10^{10} times for each cell and estimate the free area, which should be identical to $Q(l, \sigma)$.

Once the partition function Q is obtained for each cell, one can estimate the free energy per site and the chemical potential. In case of the superlattice phases, there are only two types (I and II) cells. Therefore, F_S and $\mu_{A,S}$ can be written as

$$F_{\mathrm{S}} = -k_{\mathrm{B}}T \ln \frac{N_{\mathrm{S}}!}{N_{\mathrm{A,S}}!N_{\mathrm{B,S}}!} {Q_{\mathrm{I}}}^{N_{\mathrm{S}}P_{\mathrm{I}}} {Q_{\mathrm{II}}}^{N_{\mathrm{S}}P_{\mathrm{II}}} \quad (6)$$

$$\mu_{\mathrm{A,S}} = \frac{\partial F_{\mathrm{S}}}{\partial N_{\mathrm{A,S}}} = {P_{\mathrm{I}}}^{-1} k_{\mathrm{B}}T (P_{\mathrm{I}} \ln P_{\mathrm{I}} + P_{\mathrm{II}} \ln P_{\mathrm{II}} - P_{\mathrm{I}} \ln Q_{\mathrm{I}} - P_{\mathrm{I}} \ln Q_{\mathrm{II}}) \quad (7)$$

where P_{I} and P_{II} are the fraction of cells I and II in the superlattice phase, respectively. Then, $(P_{\mathrm{I}}, P_{\mathrm{II}})$ = (1/3, 2/3) and (1/4, 3/4) for AB_2 and AB_3 superlattice phases, respectively. $N_{\mathrm{A,S}}$ and $N_{\mathrm{B,S}}$ denote the number of A and B discs in the superlattice, respectively. $N_{\mathrm{S}} = N_{\mathrm{A,S}} + N_{\mathrm{B,S}}$. k_{B} and T are the Boltzmann constant and temperature, respectively. For the random phase, we invoke the mean-field approximation that the probability for a certain type of disc to be placed next to the disc at the center would be identical to the mole fraction of the disc. We calculate all the values of 128 partition functions and the fraction of each of 128 cells based on the mean-field approximation. Because the fraction of each cell changes with x_{R}, the chemical potential $\mu_{\mathrm{A,R}} = \frac{\partial F_{\mathrm{R}}}{\partial N_{\mathrm{A,R}}}$ is also obtained as a function of x_{R} by differentiating F_{R},

$$F_{\mathrm{R}} = -k_{\mathrm{B}}T \ln \frac{N_{\mathrm{R}}!}{N_{\mathrm{A,R}}!\,N_{\mathrm{B,R}}!} \prod_{k=1}^{128} {Q_k}^{N_{\mathrm{R}}P_k}$$

$$= N_{\mathrm{R}} k_{\mathrm{B}} T \left\{ x_{\mathrm{R}} \ln x_{\mathrm{R}} + (1 - x_{\mathrm{R}}) \ln(1 - x_{\mathrm{R}}) - \ln \prod_{k=1}^{128} {Q_k}^{P_k} \right\} \quad (8)$$

where $N_{\mathrm{A,R}}$ and $N_{\mathrm{B,R}}$ denote the number of A and B discs in the random phase, respectively ($N_{\mathrm{R}} = N_{\mathrm{A,R}} + N_{\mathrm{B,R}}$). Since superlattice phases may coexist with the random phase only when the chemical potentials of two phases are equal to each other, we determine the values of x_{R} by solving $\mu_{\mathrm{A,S}} = \mu_{\mathrm{A,S}}(x_{\mathrm{R}})$ numerically. ϕ is determined from $\phi = \frac{x - x_{\mathrm{S}}}{x_{\mathrm{R}} - x_{\mathrm{S}}}$. Then, the free energy is calculated by using the equation, $F = (1 - \phi)F_{\mathrm{S}} + \phi F_{\mathrm{R}}(x_{\mathrm{R}})$. Finally, we compare the free energies of three phases: (1) AB_2 superlattice + random coexisting phase, (2) AB_3 superlattice + random coexisting phase, and (3) only random phase (Fig. 8a) and draw the theoretical phase diagram (Fig. 8b). The theoretically calculated area fractions of AB_2 superlattice (1-ϕ) and random phase (ϕ) vs. the mole fraction of A are shown in Supplementary Fig. 6a. When the mole fraction of A is small, only the random phase exists. As the mole fraction A is increased further the fraction of AB_2 phase increases linearly while the fraction of random phase decreases. When the mole fraction of A becomes 0.33, a pure

AB_2 phase is formed, after which the fraction of AB_2 phase decreases while the fraction of random phase increases.

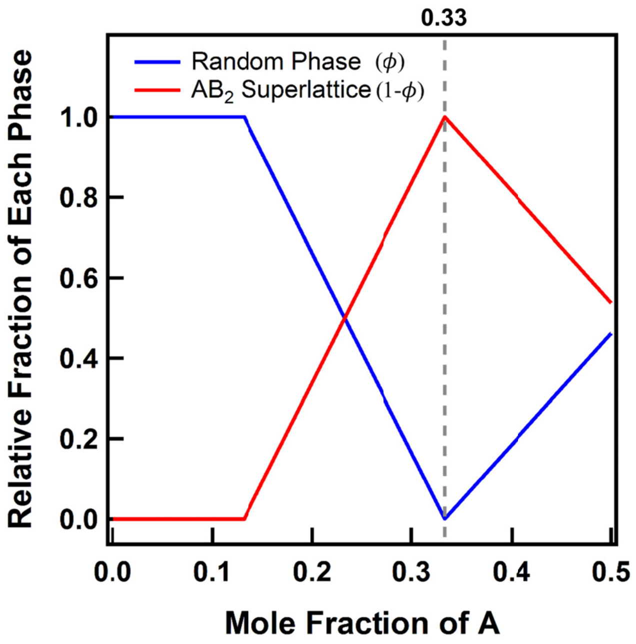

Supplementary Figure 6. Theoretically calculated relative areal fractions of AB_2 and R phases vs. the mole fraction of particle A. In this calculation, the particle size ratio of 0.2 is used.

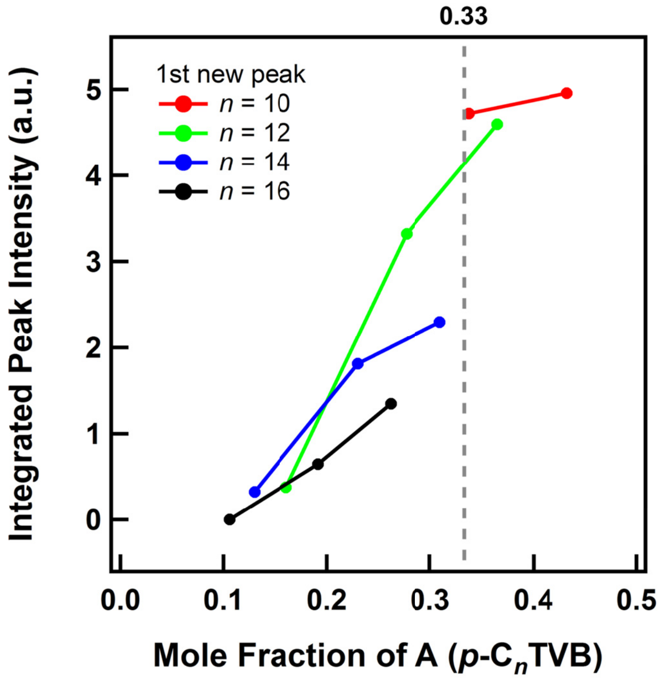

Supplementary Figure 7. Integrated intensity of the 1st new peak as a function of mole fraction of A. Integrated intensities of the 1st new peak (q=0.75 nm^{-1}, for AB_2 superlattice) at 6 °C vs. the mole fraction of A (p-C_nTVB) for all different n. The integrated intensities of the 1st new peak monotonically increase with the mole fraction of A until the mole fraction of A becomes 0.33, indicating that the fraction of AB_2 superlattice relative to the random phase increases. This is consistent with the theoretically calculated fraction of the AB_2 phase as shown in Supplementary Fig. 6. Above 0.33, the increase of intensity is significantly suppressed or saturated. This change in intensity variation across 0.33 is consistent with the theoretical calculation although the measured intensity does not show the decrease in intensity as expected from the theoretical calculation.

Supplementary References

1. Guinier, A.; Fournet, G. *Small-Angle Scattering of X-Rays*. (John Wiley and Sons, 1955).
2. Feigin, L. A. & Svergun, D. I. *Structure Analysis by Small-Angle X-Ray and Neutron Scattering*. (Springer US, 1987).
3. Higgins, J. S. & Benoît, H. *Polymers and neutron scattering*. (Clarendon press Oxford, 1994).
4. Selinger, J. V. & Bruinsma, R. F. Hexagonal and nematic phases of chains. I - Correlation functions. II - Phase transitions. *Phys. Rev. A* **43,** 2910–2921 (1991).
5. Schmidt, P. W. Small angle X-ray scattering from helical filaments. *J. Appl. Crystallogr.* **3,** 257–264 (1970).
6. Hamley, I. W. Form factor of helical ribbons. *Macromolecules* **41,** 8948–8950 (2008).
7. Wheatley, R. J. Phase diagrams for hard disc mixtures. *Mol. Phys.* **93,** 965–969 (1998).
8. Wang, Y. L., Ree, T., Ree, T. S. & Eyring, H. Significant-structure theory and cell theory for two-dimensional liquids of hard disks. *J. Chem. Phys.* **42,** 1926–1930 (1965).
9. Cottin, X. & Monson, P. a. A cell theory for solid solutions: Application to hard sphere mixtures. *J. Chem. Phys.* **99,** 8914–8921 (1993).
10. Cottin, X. & Monson, P. A. Substitutionally ordered solid solutions of hard spheres. *J. Chem. Phys.* **102,** 3354–3360 (1995).
11. Widom, B. Some Topics in the Theory of Fluids. *J. Chem. Phys.* **39,** 2808–2812 (1963).
